# Multiplexed Liquid Biopsy and Tumor Imaging Using Surface-Enhanced Raman Scattering

**DOI:** 10.3390/bios11110449

**Published:** 2021-11-12

**Authors:** Francesco Dell’Olio

**Affiliations:** Department of Electrical and Information Engineering, Polytechnic University of Bari, 70125 Bari, Italy; francesco.dellolio@poliba.it

**Keywords:** biophotonics, biosensing, nanoplasmonics, surface-enhanced Raman scattering, oncology

## Abstract

The recent improvements in diagnosis enabled by advances in liquid biopsy and oncological imaging significantly better cancer care. Both these complementary approaches, which are used for early tumor detection, characterization, and monitoring, can benefit from applying techniques based on surface-enhanced Raman scattering (SERS). With a detection sensitivity at the single-molecule level, SERS spectroscopy is widely used in cell and molecular biology, and its capability for the in vitro detection of several types of cancer biomarkers is well established. In the last few years, several intriguing SERS applications have emerged, including in vivo imaging for tumor targeting and the monitoring of drug release. In this paper, selected recent developments and trends in SERS applications in the field of liquid biopsy and tumor imaging are critically reviewed, with a special emphasis on results that demonstrate the clinical utility of SERS.

## 1. Introduction

When photons interact with matter, several scattering phenomena, either elastic or inelastic, can occur. From the application point of view, Raman scattering [1,2] is the most attractive inelastic scattering phenomenon, since it is widely used to characterize organic and inorganic samples by monitoring the intensity and wavelength of light inelastically scattered from the atoms/molecules forming a sample.

**Figure 1:**
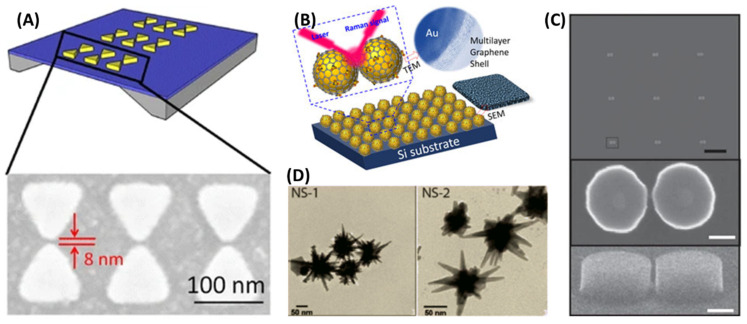
Examples of SERS substrates. (**A**) Au bowtie nanoantenna array deposited on a Si_3_N_4_ membrane. (**B**) Graphene-encapsulated AuNPs on Si substrate. (**C**) Array of Si dimer nanoantennas: SEM image of the array of nanostructures, SEM top-view and lateral-view images of a single nanoantenna. (**D**) Gold nanostars with different spike morphologies. SEM: scanning electron microscopy. TEM: transmission electron microscopy. Reproduced with permission from [7,8,9]. Copyright 2015, Springer. Copyright 2015, Nature Publishing Group. Copyright 2014, Royal Society of Chemistry.

Raman scattering has a key issue—the extremely small cross-section of the Raman process, which is 12–14 orders of magnitude below the cross-section of fluorescence. In order to overcome this crucial drawback, samples can be placed on a nanostructured surface to enhance the Raman signal by a factor of up to 10^16^ according to two mechanisms: electromagnetic enhancement due to optical field confinement in nanometer-scale regions called hot spots and chemical enhancement, which refers to contributions to Raman scattering that do not rely on the spatial distribution of the electromagnetic field [3,4,5]. Due to its evident advantages over Raman scattering, SERS has become a powerful tool in biophysics/biochemistry and life sciences. The performance of SERS-based systems critically depends on the features of SERS-active substrates, which are mostly—but not exclusively—metallic platforms consisting of either periodic planar nanostructures with two-dimensional periodicity, such as plasmonic nanoantenna arrays, or colloidal nanoparticles (NPs) in solution—e.g., quasi-spherical Ag/AuNPs, Au nanorods/nanostars, and NP aggregates (see Figure 1) [6,7,8,9]. Recent emerging experimental evidence indicates that dielectric metasurfaces have the potential to produce the SERS enhancement of an extent equivalent to that of plasmonic substrates [10], but further studies are required before the use of dielectric/semiconducting substrates in SERS experiments becomes widespread.

The life sciences applications of SERS are almost exclusively related to the detection of target molecules, macromolecules, cells, and microorganisms in samples with different levels of complexity according to two basic methodological approaches: label-free detection and indirect detection [11]. The former does not require any labeling activity on the sample but provides complex spectra that may be challenging to interpret, while the latter implies the use of the so-called SERS tags, Raman reporter molecules emitting strong and distinct Raman signals.

Since SERS was accidentally discovered in 1974, its application has increased enormously, but medicine still remains the most promising domain for its application. In fact, SERS is a well-established analytical technique with many advantages over other competing techniques. It possesses unique features, such as its sensitivity and capacity for multiplexing, that are not found in other approaches, including enzyme-linked immunosorbent assay (ELISA), polymerase chain reaction (PCR), fluorescence in situ hybridization (FISH), fluorescence, and flow cytometry. The critical aspects of SERS-based approaches for the quantitative detection of biomarkers are related to their reproducibility, but several recent studies have addressed and analyzed the sources of this irreproducibility in order to show how these can be minimized [12].

The medical applications of SERS are generally related to the emerging paradigm of precision and personalized medicine [13,14,15,16]. They include the detection of pathogens/biomarkers correlated with different pathologies and tissue imaging during several diagnostic/surgical procedures.

According to a very recent report of the World Health Organization, in 134 out of 183 countries, cancer is the first or second leading cause of premature death at ages 30–69 years, responsible for nearly 10 million deaths in 2020. In addition, cancer has a significant and increasing economic impact, with an annual cost exceeding USD 1 trillion [17]. The medical approach to managing this pathology is quickly changing, moving toward new personalized and proactive paradigms that include the use of biomarkers and advanced imaging approaches. In this rapidly changing context, early diagnosis and therapy for tumors are becoming increasingly challenging, requiring sophisticated diagnostic tools that are able to detect the disease early, fully characterize the tumor, and monitor its progression.

SERS detection has been extensively studied in relation to its application in oncology [18,19,20,21,22,23], with the findings of these activities suggesting that the features of SERS are very useful for tumor imaging and fit the needs of precision oncology—evidence-based individualized medicine that aims to deliver the right care to the right cancer patient at the right time—very well.

In this paper, the recent advances in the field of in vitro and in vivo SERS applications in oncology are reviewed. In addition, several studies relating to the clinical utility of SERS-based technologies are discussed. The paper concludes with some notes on the future prospects for this technology.

## 2. In Vitro Cancer Biomarker Detection

Liquid biopsy, the in vitro detection of tumor-derived biomarkers in body fluids (blood, cerebrospinal fluid, urine, sputum, and ascites), is a promising technique in diagnosis, with some evidence of its clinical utility for a wide range of diagnostic applications, including in the identification of drug resistance mechanisms, patient stratification, the prediction of treatment efficacy, and the identification of drug resistance mechanisms [24,25,26,27].

Many circulating tumor-derived factors, including circulating tumor cells (CTCs), cell-free tumor DNA (ctDNA), cancer-derived exosomes, mRNA, cell-free microRNAs (cfmiRNAs), long non-coding RNA, small RNA, circulating cell-free proteins, and tumor-educated platelets, have been identified as a result of extensive research over the last few decades [28]. In addition, numerous technologies for their detection have been developed. The most recent advances in the field of liquid biopsy are reviewed in [29].

The potential of both label-free and indirect SERS-based technologies has been extensively studied, with the goal of developing novel, reliable, and clinically useful approaches for analyzing samples where circulating tumor-derived factors are dispersed in order to improve the state-of-the-art in the technologies used for liquid biopsies.

### 2.1. CTCs Quantitative Detection

CTCs are a rare subset of cells (with only 1–10 CTCs found among around 10 million leukocytes and 5 billion erythrocytes in 1 mL of blood) that can be found in the blood of patients with solid tumors since they are released by primary tumors and/or metastatic sites. Many clinical studies have concluded that CTC number is an important indicator of the risk of progression or death in patients with metastatic and localized solid cancer (e.g., breast, prostate, and colon) [30,31].

More than one decade after the pioneering work by Sha et al. [32], the potential of SERS spectroscopy for the quantitative detection of CTCs is now well established, and limit of detection (LoD) values down to 1 cell/mL have been experimentally demonstrated.

**Figure 2:**
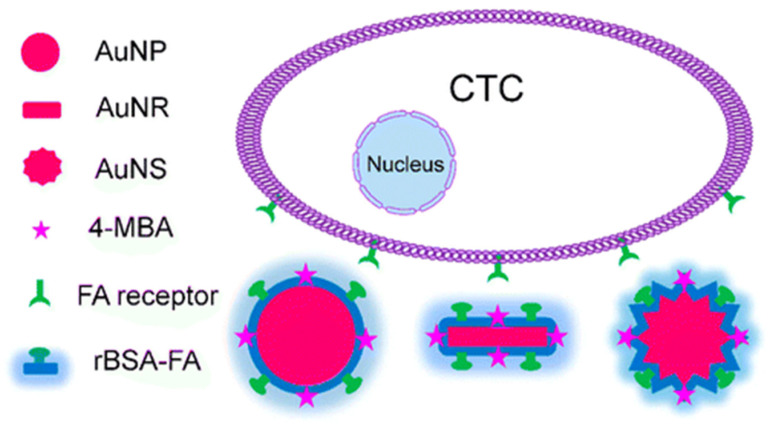
SERS-active NPs of various shapes used in CTC detection: spherical gold nanoparticles (AuNPs), gold nanorods (AuNRs), and gold nanostars (AuNSs). These NPs possess a strong SERS signal due to the modification of 4-mercaptobenzoic acid (4-MBA), which is the Raman reporter molecule. rBSA: reductive bovine serum albumin, which reduces nonspecific catching or uptake by healthy cells in the blood. FA: folic acid, targeted ligand. Reproduced with permission from [33]. Copyright 2016, American Chemical Society.

A very interesting aspect related to the use of SERS spectroscopy in quantitative CTC detection is that this technique does not necessarily require an enrichment step, as is required by the majority of CTC detection techniques due to the scarcity of CTCs in peripheral blood. One of the recent key achievements in this key aspect is the demonstration of SERS-active NPs for CTC detection without an enrichment process and with an LoD of 1 cell/mL [33]. To achieve this result, three kinds of SERS-active NPs with similar particle sizes, similar modifications, and different shapes (spherical NPs, nanorods, and nanostars) have been developed (see Figure 2). For all NPs, reductive bovine serum albumin has been used to encapsulate AuNPs, and folic acid was conjugated to the surface of these NPs to recognize CTCs. The best or lowest LoD value, indicating the highest sensitivity, was found for Au nanostars.

**Figure 3:**
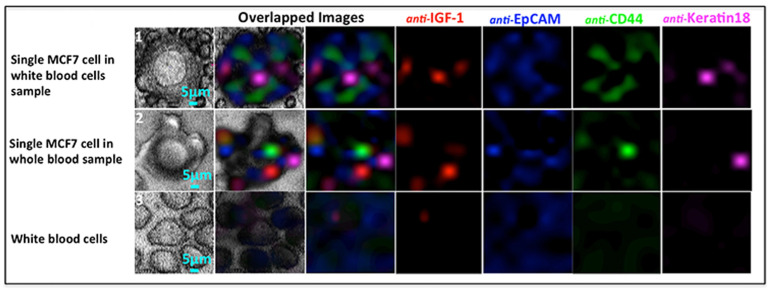
Multicolor SERS analysis of MCF-7 cells in blood: (**1**) a single MCF-7 cell among white blood cells; (**2**) a single MCF-7 cell in whole (unprocessed) blood; (**3**) white blood cells only. Reproduced with permission from [37]. Copyright 2016, Nature Publishing Group.

Scarcity is not the only issue encountered in the quantitative detection of CTCs. In fact, several experimental results have shown that the use of a single CTC marker, typically the epithelial cell adhesion molecule (EpCAM), a cell-surface transmembrane glycoprotein, can lead to false positive/false negative results [34]. Thus, specific panels of highly specific markers should be utilized for the accurate discrimination of CTCs. This need is surely compliant with the basic features of SERS spectroscopy, and the multiplexed detection of CTCs has been investigated in the last few years by several research groups. In particular, SERS was demonstrated to enable very accurate discrimination of CTCs from other cells when using up to five recognition ligands [35,36]. Nima et al. reported some of the most interesting results in the field of the multiplexed detection of CTCs based on SERS [37]. Ag–Au nanorods have been functionalized with four different Raman-active molecules and conjugated with four kinds of antibodies (anti-epithelial cell adhesion molecule (anti-EpCAM), anti-CD44, anti-keratin, and an anti-insulin-like growth factor antigen (anti-IGF-I receptor β)) specific to breast cancer markers. In addition, SERS detection has been combined with photothermal resonance detection. The detection of single breast cancer cells in unprocessed human blood has been demonstrated. In particular, these experiments have proven that just a single target MCF7 CTC molecule can be successfully detected in 1 million red blood cells; see Figure 3.

The phenotypic characterization of CTCs is critical for monitoring disease progression during pharmacologic treatment. It was recently established that SERS may be used to characterize the phenotypic evolution of cells. A fascinating study investigated stage IV melanoma patients who were receiving molecular targeted or immunological treatments. The findings suggest that the SERS-based method reported in [38] may be able to effectively define phenotypic alterations in CTCs from these patients. This technique uses AuNPs with a diameter of 60 nm that are antibody-conjugated and Raman reporter-coated and includes multiple steps, the first of which is centrifugation and CD45 depletion to remove red blood cells and leukocytes, respectively. The remaining cells are then incubated with the four distinct antibody-conjugated SERS labels, followed by Raman spectroscopy detection. This approach can identify 10 cells in 10 mL of blood, has the advantage of not requiring CTC enrichment beforehand, and can be highly multiplexed, since several surface protein expression profiles can be simultaneously monitored.

### 2.2. Exosome Detection

Exosomes are a subgroup of extracellular vesicles with a diameter in the range of 30–150 nm that have been linked to numerous processes associated with cell-to-cell communications, such as cell proliferation, cell migration, cancer metastasis, and immunomodulatory activity [39]. The key role of tumor-derived exosomes in cancer development, metastasis, immune response regulation, and the induction of angiogenesis is well established [40]. Thus, the potential of exosomes as promising biomarkers for liquid biopsies has been widely investigated in recent years.

Exosome detection usually requires an initial isolation step. The gold standard for purifying exosomes is the ultracentrifugation protocol reported by Thery et al. in 2006 [41]. ELISA, flow cytometry, and nanoparticle tracking analysis are the most common techniques used for characterizing exosomes after purification, evaluating the number of exosomes and/or the expression levels of disease-related proteins. Due to the abovementioned limitations of these techniques, new approaches, including SERS-based ones, are currently under development.

Research studying the possibility of using SERS for analyzing exosomes started just a few years ago but is already showing very interesting results. In particular, some recent findings confirm that properly engineered SERS tags or label-free approaches can be utilized for both the detection and phenotypic profiling of tumor-derived exosomes [42,43,44,45].

**Figure 4:**
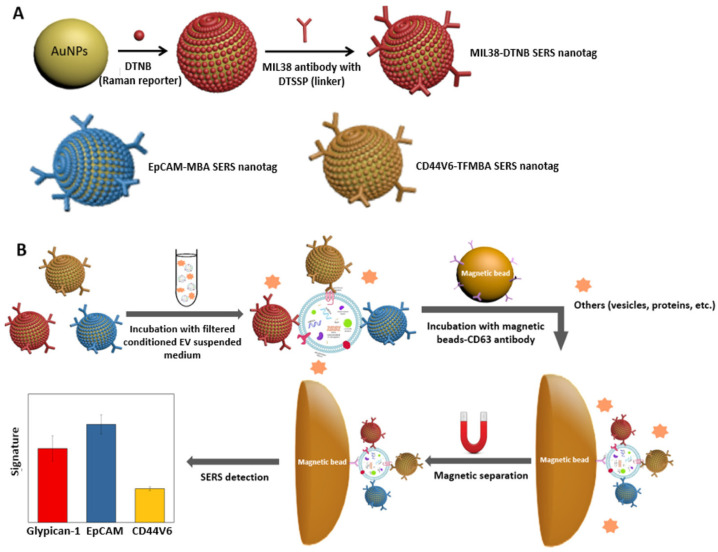
SERS-based sandwich immunoassay for the phenotypic profiling of cancer-derived small extracellular vesicles. (**A**) Nanotag preparation. (**B**) SERS nanotags and magnetic beads used for the molecular phenotype profiling of CD63-positive extracellular vesicles (EVs). Reproduced with permission from [46]. Copyright 2020, American Chemical Society.

The sandwich immunoassay for the fast and multiplexed phenotypic profiling of exosomes released by a human pancreatic cancer cell line (Panc-cells) (published in [46]) is an example of a technique based on SERS tags. This assay combines antibody-coated SERS nanotags and magnetic beads for magnetic separation, eliminating the need for complicated isolation processes. SERS nanotags are AuNPs that are covalently linked to Raman reporter molecules. For this, three biomarkers are typically selected, namely Glypican-1, EpCAM, and CD44 variant isoform 6, whereby antibodies used for their selective recognition have been conjugated on the NP surface. As summarized in Figure 4, the assay, which has an LoD in the order of 10^6^ exosomes per mL, includes two incubation steps. NPs are disseminated in the medium in which exosomes were suspended during the first incubation step, while functionalized magnetic beads are used in the second. For phenotypic profiling, a portable Raman spectrometer was utilized. This excites samples with a laser beam at 785 nm (power = 15 mW) and Raman spectra are acquired with an integration time of 10 s.

Very recently, an ultrasensitive exosome detection method with a record LoD of 2.4 exosomes/μL was reported [47]. In mice models, developed SERS aptasensors, which were designed for use in postoperative recurrence surveillance, have shown high sensitivity in identifying tumors with an average diameter of 3.55 mm.

As mentioned, label-free SERS detection of exosomes is also possible, but because of the complexity of biological samples, complicated spectra are obtained and should be analyzed and interpreted by comparing the SERS spectra of exosomes from cancer patients to those of healthy people [48]. To identify tumor-specific spectral signatures with a high degree of accuracy, complicated statistical methods, such as principal component differential function analysis, are widely used. Carmicheal et al., for example, demonstrated an approach that had 90% accuracy in distinguishing pancreatic cancer exosomes from exosomes of normal pancreatic epithelial cell lines [49]. This methodology is based on SERS measurements that are carried out using a confocal Raman microscope at 785 nm (10 mW laser power).

### 2.3. Detection of ctDNA and cfmiRNA

Apoptotic or necrotic cancer cells actively release a class of circulating cell-free DNA (ccfDNA), called ctDNA, into the bloodstream (or another biological fluid) [50,51,52,53]. ctDNA comprises only a small fraction (<1%) of all ccfDNA because most ccfDNA comes from normal cells under physiological conditions. Thus, very sensitive and specific strategies are required to distinguish rare ctDNA from normal ccfDNA.

Currently, the most well-established technologies for ctDNA analysis are either targeted or untargeted [54,55,56,57,58,59,60]. Targeted approaches are obviously more sensitive than untargeted ones and can detect only specific known somatic mutations/epigenetic alterations that have been discovered in a primary tumor. They include digital PCR, cancer personalized profiling by deep sequencing (CAPP-Seq), BEAMing (beads, emulsion, amplification, magnetics) technology, the safe-sequencing system (Safe-SeqS), and tagged-amplicon deep sequencing (TAmSeq). For the genome-wide detection of copy number aberrations, point mutations, and/or other genetic aberrations, untargeted sequencing is used.

At present, only a few promising SERS ctDNA detection results have been reported. ctDNA detection by enzymatic amplification paired with SERS tagging was established with femtomolar and subfemtomolar LoD values [61,62]. Recently, a novel test combining SERS and PCR that can identify three clinically relevant melanoma DNA alterations in ctDNA was developed [63]. Experiments have shown that the sensitivity and accuracy of this method are comparable to those of droplet digital PCR.

CfmiRNAs are fragments of single-stranded noncoding RNA comprising 19–25 nucleotides. A wide range of experimental findings demonstrate that many miRNAs are involved in several types of cancers, playing key roles in tumorigenesis, progression, and metastasis [64,65].

After demonstrating that SERS tests with properly engineered tags are effective instruments for miRNA detection and classification [66], a complex core–satellite nanostructure with a plasmonic Au nanodumbbell as the core and AuNPs as satellites [67] demonstrated a record LoD of 0.85 aM. According to an “off-to-on” SERS method, the target miRNA (miRNA-1246) triggers the assembly of nanostructures and turns on the SERS signal.

**Figure 5:**
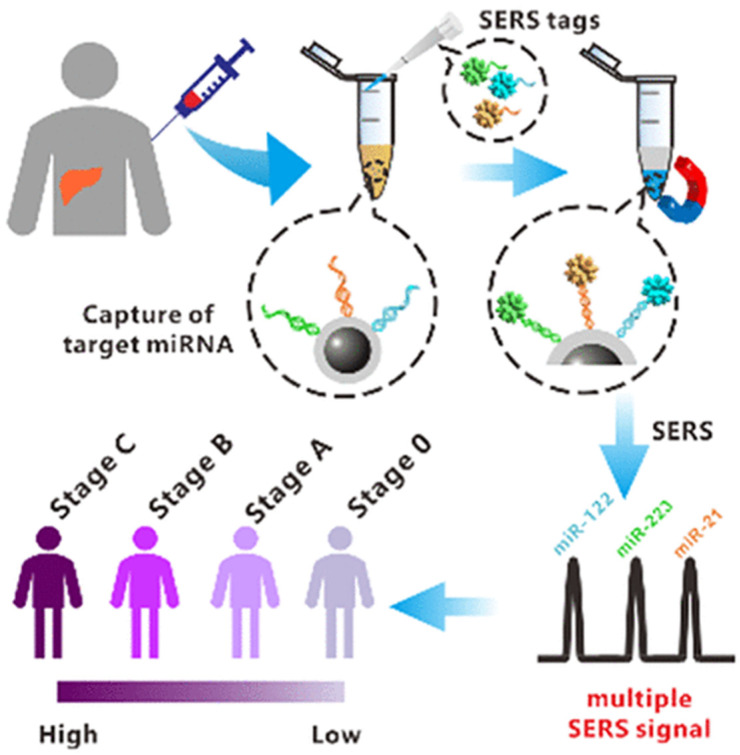
Schematic illustration of the use of a SERS-based method for the early diagnosis and prognosis of hepatocellular carcinoma. Reproduced with permission from [68]. Copyright 2021, American Chemical Society.

The multiplex detection of three hepatocellular carcinoma-related miRNA (miRNA-122, miRNA-21, and miRNA-223) biomarkers using magnetically assisted sandwich-type SERS was recently demonstrated [68]. The detection is based on Raman dye-modified fractal AuNPs and Ag-coated magnetic NPs. A LoD of 311 aM was achieved for miRNA-21, 349 aM for miRNA-122, and 374 aM for miRNA-223. The potential of this method in staging hepatocellular carcinoma patients has been widely proven, as shown in Figure 5.

### 2.4. Detection of Cancer-Related Proteins

Blood- or urine-based tests for detecting abnormal levels of cancer-related proteins—e.g., carcinoembryonic antigen, prostate-specific antigen (PSA), and alpha-fetoprotein—have been used routinely for several years in the early diagnosis and monitoring of tumors [69,70]. Panels of blood protein markers have been applied in early diagnostics, the monitoring of cancer recurrence, and for predicting therapeutic response. For example, the prostate health index (PHI) is a well-established blood-based test that provides a probability of prostate cancer by combining three tests (PSA, free PSA, and p2PSA) into a single score [71].

In clinical practice, the identification and quantification of protein markers, which are often of low abundance in blood, is carried out using immunological techniques, including enzyme-linked immunosorbent assays or Western blotting, but several studies have demonstrated the superiority of using SERS-based approaches, especially in terms of their sensitivity, LoD, and multiplexing.

Experiments have been conducted on a variety of SERS-based approaches, including those that use the dissociation of core–satellite assemblies to turn SERS signals “off” or the development of a sandwich structure to generate a SERS signal.

**Figure 6:**
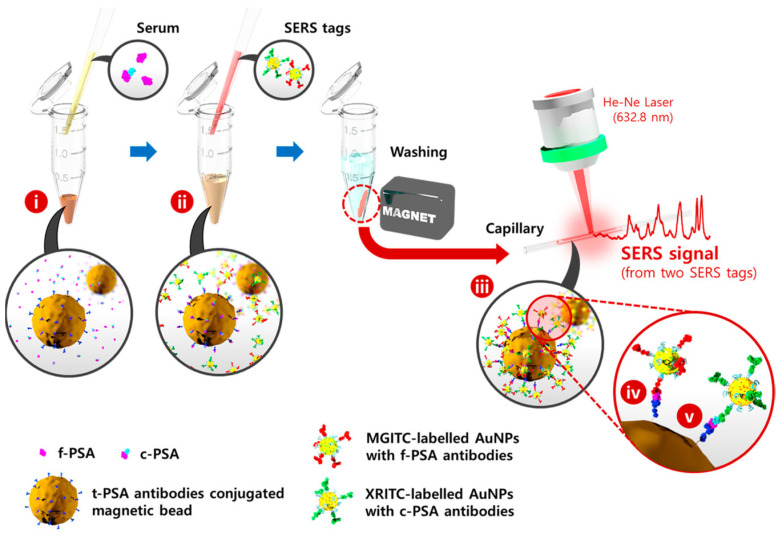
SERS-based assay for the simultaneous detection of f-PSA and c-PSA. MGITC and XRITC are the Raman reporter molecules. MGITC: malachite green isothiocyanate. XRITC: X-rhodamine-5-(and-6)-isothiocyanate. Reproduced with permission from [73]. Copyright 2017, American Chemical Society.

A SERS-based immunosensor with a LoD value of 7 fg/mL has been effectively used for the detection of vascular endothelial growth factor in human blood plasma taken from patients with breast cancer [72]. More recently, a SERS-based immunoassay for the simultaneous detection of dual prostate-specific antigens has been reported [73]. This method uses SERS nano tags and magnetic beads to estimate the free to total PSA ratio. In a microtube, serum containing both free and complexed PSA antigens, f-PSA and c-PSA, is combined with total-PSA-conjugated magnetic beads, as shown in Figure 6. Both antigens are trapped on the surface of magnetic beads at this point and labeled AuNPs are then added to form immunocomplexes. In this manner, magnetic immunocomplexes may be separated using a magnetic bar and the Raman signals for each SERS tag can be detected and analyzed. The assay results have been found to very closely match those obtained using a standard electrochemiluminescence system.

## 3. In Vivo Imaging

The basic function of oncological imaging [74,75] is to detect tumor tissue and differentiate it from normal tissue. In addition, imaging is very often used to assess the efficacy of anticancer treatments, such as chemotherapy or radiotherapy.

Each oncological imaging technique, including the most well-established ones, such as magnetic resonance imaging and computed tomography, basically detects changes within tissues, or cells when a tumor forms. The understanding of the fundamental tissue, cellular, and molecular changes that form the hallmarks of cancer is now rather advanced. Consequently, very sophisticated imaging techniques that aim at detecting cancers in their earliest stages are currently under development [76].

Optical imaging, which is a non-invasive and non-ionizing technology, has very good spatial resolution down to the nanometer range and is able to provide quantitative and real-time information. Thus, several promising optical imaging techniques are currently being explored in the framework of in vivo imaging studies in animals [77,78].

**Figure 7:**
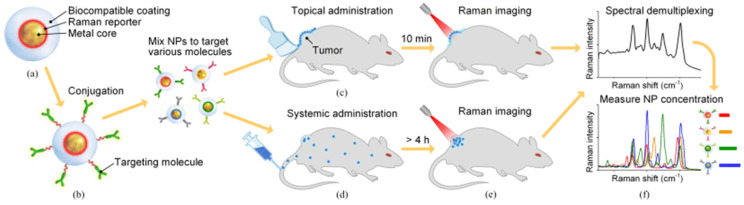
SERS NPs and their application in oncological SERS imaging. (**a**) Typical sandwich structure of SERS NPs. (**b**) SERS NPs functionalization. Operating principle of SERS imaging: (**c**) topical administration of SERS NPs, (**d**) systemic administration of SERS NPs, (**e**) imaging, and (**f**) demultiplexing. Reproduced with permission from [79]. Copyright 2016, IEEE.

At present, the tremendous potential of SERS in the field of emerging oncological imaging techniques is widely recognized due to the extreme sensitivity of this methodology. SERS in vivo imaging is based on SERS NPs, which typically have a sandwich structure. Figure 7 shows the typical sandwich structure of SERS NPs and the operating principle of SERS imaging for tumor detection [79]. These NPs have a metal core. A layer of Raman reporter molecules is permanently adsorbed onto the core’s surface, with the goal of triggering the SERS effect, which involves the formation of a particular Raman spectrum in response to laser excitation at a specific wavelength. As different “flavors” of SERS NPs use distinct reporter molecules to generate specific spectral signatures, the resultant SERS spectrum acts as a fingerprint that allows the identification of that particular “flavor” of NP. A biocompatible covering surrounds the NP, stabilizing the SERS NP signal. SERS NPs, when employed for biomolecule detection, can be conjugated to a molecule, such as an antibody or affibody to precisely target the biomolecule of interest, such as epidermal growth factor receptor (EGFR) or human epidermal growth factor receptor 2 (HER2).

Research in the field of SERS-based cancer in vivo imaging began approximately one decade ago with two seminal papers clearly demonstrating the potential of this approach [80,81]. The paper by Quian et al. [80] demonstrated that EGFR-positive tumor xenografts in animal models can be targeted and detected in vivo using SERS and biocompatible/nontoxic gold NPs with single-chain variable fragment antibodies as tumor-targeting ligands, organic dyes as reporters, and thiol-modified polyethylene glycols as stabilizers. The paper by Keren and co-workers [81] reported the first SERS image of a whole organ (liver) in a living mouse. A microscopic table equipped with a horizontal translation stage was utilized for raster scanning and Raman imaging of the anesthetized mouse; after that, SERS tags—commercial glass-coated AuNPs functionalized with Raman reporter molecules—were injected into its tail vein. SERS images acquired 2 h after injection revealed a bright liver due to NPs being naturally taken up by the reticuloendothelial system. The maximum penetration depth of the Raman microscope was estimated to be equal to 5.5 mm if the concentration of SERS NPs is low (1.3 nM). The paper also demonstrated the multiplexing potential of SERS imaging. In fact, the imaging system was able to rapidly and straightforwardly distinguish four types of SERS NPs presenting different Raman spectra.

**Figure 8:**
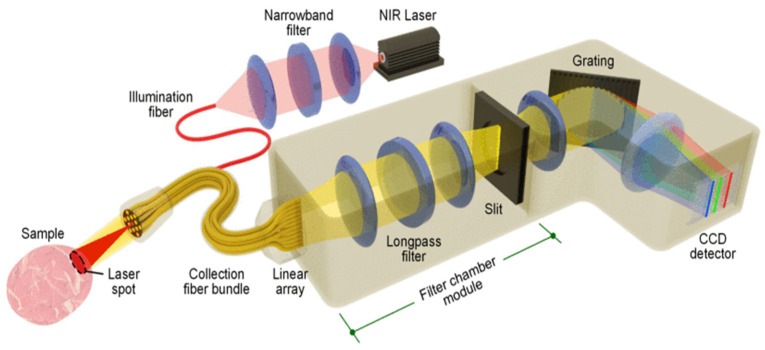
Typical experimental setup operating at NIR for SERS NP imaging in tissues. Reproduced with permission from [79]. Copyright 2016, IEEE.

After these pioneering achievements, much progress has been demonstrated with respect to the specificity, penetration depth, multiplexing capability, and potential oncological applications of SERS imaging [82,83,84,85]. Although the first experiments were carried out in the visible range, the wavelength most used in SERS imaging at present is 785 nm, which corresponds to near infrared (NIR), and allows improved penetration depth of the technique. The typical configuration of the experiment setup utilized in SERS imaging is shown in Figure 8. In this setup, an NIR laser generates the lightwave for sample excitation, and light from the tissue is collected by multiple fibers in the imaging probe and delivered through a fiber bundle to a spectrometer, including a charge-coupled device (CCD) detector. The current maturity of SERS imaging technology allows its use as a very effective tool for the imaging of surgical margins to guide the complete removal of tumors [79,86,87,88,89].

Demonstrating the potential medical applications of SERS imaging in animal models is currently a topic of intense research efforts. For example, a miniature spectral endoscope for the rapid SERS imaging of multiplexed receptor-targeted SERS NPs was reported in [90]. This paper demonstrates the potential ability of the endoscope to detect esophageal tumors. A cocktail of receptor-targeted SERS NPs was topically applied to the luminal surface of a rat esophagus to target EGFR and human HER 2. Then, the lumen of the esophagus was comprehensively imaged to identify the NPs bound to it. The visualization of tumor locations and the quantification of biomarker expression were demonstrated using this approach.

**Figure 9:**
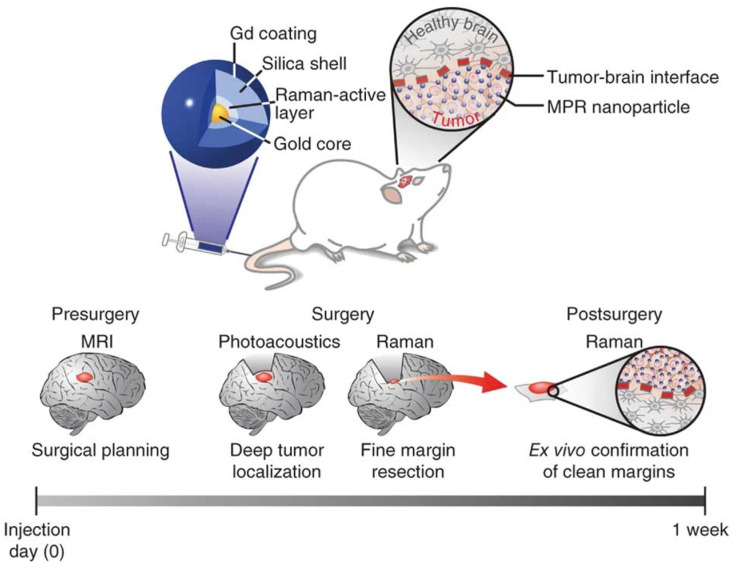
Imaging strategy based on MPR NPs. Gd: gadolinium. Reproduced with permission from Ref. [91]. Copyright 2016, Nature Publishing Group.

Some very interesting studies concerning the possibility of integrating SERS with other imaging modalities are available in the literature. A high-potential brain tumor molecular imaging strategy based on magnetic resonance, photoacoustics, and SERS was reported by Kircher et al. [91] (see Figure 9). Very sophisticated magnetic–photoacoustic–Raman (MPR) NPs were engineered, synthesized, and intravenously injected into a mouse bearing an orthotopic brain tumor. The ability of MPR NPs to be detected by magnetic resonance imaging allows for tumor identification and surgical planning prior to surgery. Photoacoustic imaging can then guide bulk tumor excision intraoperatively due to its relatively high resolution and deep tissue penetration. Finally, SERS imaging can be employed to remove any remaining microscopic tumor burden due to its ultrahigh sensitivity and spatial resolution. The Raman probe can be utilized ex vivo to examine the resected specimen in order to verify clear tumor margins.

## 4. Conclusions and Future Perspectives

The critical analysis of results in this review paper unequivocally demonstrates the potential of the use of SERS technology in oncology. Although the results were largely obtained from spiked samples and animal models, they clearly demonstrate that the physical effect of SERS enables high-sensitivity liquid biopsies capable of identifying tumor markers with a high predictive capacity, also highlighting that the usefulness of applying sophisticated imaging techniques in the intraoperative phase.

Since there are very few registered and published clinical trials concerning the use of SERS spectroscopy in oncology [92], it is unlikely that this technology will be used in clinical practice in the near future. In spite of the considerable research efforts carried out, especially in the last decade, SERS-based methodologies have a level of repeatability that is not compatible with clinical practice. Furthermore, the instrumentation used for SERS spectroscopy is still not miniaturized, is usable only by experts in the field, and is not automated. These critical aspects could be partially overcome by using new homogeneous SERS substrates fabricated with CMOS-compatible techniques, such as metasurfaces, as well as by evaluating the use of nanoscale optical trapping techniques in the context of SERS technologies. In addition, user-friendly, portable, and compact miniaturized systems for SERS spectroscopy are under development, and the achievements reported in the literature—especially regarding the combination of SERS spectroscopy and microfluidics—are highly encouraging [93]. Finally, the most recent advances in the field of machine learning and artificial intelligence are profoundly transforming the algorithms used for SERS spectra processing, leading the way to new intriguing applications of SERS in the early diagnostics and treatment of cancer.

The combination of SERS-based methodologies with other analytical and imaging techniques is an open research field. Only a few interesting achievements in this area have been recorded in the literature to date but, conceptually, it is highly probable that SERS technology will benefit from such a combination.
